# Metabolite Profiling and Classification of Developing *Styrax tonkinensis* Kernels

**DOI:** 10.3390/metabo10010021

**Published:** 2020-01-01

**Authors:** Qikui Wu, Xue Zhao, Chen Chen, Zihan Zhang, Fangyuan Yu

**Affiliations:** 1 Collaborative Innovation Center of Sustainable Forestry in Southern China, College of Forest Science, Nanjing Forestry University, Nanjing 210037, China; qkwu@njfu.edu.cn (Q.W.); zhaoxue921207@foxmail.com (X.Z.); cc0212@njfu.edu.cn (C.C.); zhangzh@njfu.edu.cn (Z.Z.); 2 Mingguang Forestry Station, Mingguang 239400, China; 3 State Key Laboratory of Tree Genetics and Breeding & Key Laboratory of Tree Breeding and Cultivation, State Forestry Administration, Research Institute of Forestry, Chinese Academy of Forestry, Haidian, Beijing 100000, China

**Keywords:** *Styrax tonkinensis*, kernel development, metabolome analysis, metabolite classification

## Abstract

Background: *Styrax tonkinensis* is an economic tree species with high timber, medicine, oil, and ornamental value. Its seed, containing a particularly high oil content, are widely studied for their biodiesel properties by nutritional components and oil body ultrastructure. However, their comprehensive biochemical compositions have not been studied. Methods: During *S. tonkinensis* kernel development, we collected samples from four time points for metabolite profiling and classification through gas chromatography-mass spectrometry and liquid chromatography-mass spectrometry. Results: A total of 187 and 1556 metabolites were obtained, respectively. All of the metabolites were grouped into 19 and 21 classes by their chemical properties and into 8 clusters based on their change trends, respectively. Among all the metabolites, carboxylic acids and derivatives, flavonoids, fatty acyls, glycerophospholipids, organooxygen compounds, prenol lipids, and steroids and steroid derivatives were the main components. Alanine, glutamine, tryptophan, tyrosine and valine were the five most abundant amino acids. Palmitic acid, stearic acid, oleic acid and linoleic acid were the four major free fatty acids. Flavans, flavonoid glycosides and o-methylated flavonoids were the three major flavonoids. The differential metabolites distributions between different time points were identified. A pathway enrichment was performed, which was mainly focused on three groups, amino acids metabolism, carbon flow from sucrose to lipid and secondary metabolites biosynthesis. Conclusions: It’s the first time to analyze the metabolite fingerprinting for developing *S. tonkinensis* kernels and identify varied kinds of flavonoids. We performed metabolite profiling, classification and pathway enrichment to assess the comprehensive biochemical compositions. Our results described the change in major metabolites and main metabolic processes during *S. tonkinensis* kernel development and provided a variety of bases for seed applications as biofuel or medicine.

## 1. Introduction

*Styrax tonkinensis* (Pierre) Craib ex Hartwich, a member of the *Styracaceae* family, has high timber, medicine, oil, and ornamental value [1,2,3]. This species has been widely cultivated for wood production as pulpwood species and benzoin resin collection used in perfumes and medicines in Laos, Vietnam and southern China [1,4]. It has also been verified as potential for biofuel with bioethanol from woody biomass and biodiesel from seeds [5,6]. The content of seed lipid for biodiesel is dependent on the carbon balance between different nutrients and the triacylglycerol accumulation in oil bodies [3,7]. The major content of *S. tonkinensis* seeds is lipid with excellent fatty acids (FA) composition, followed by protein, soluble sugar and amino acids. *S. tonkinensis* seed oil has perfect biodiesel properties [6], satisfying the standards of China (GB/T 20828), the European Union (EN 14214), Germany (DIN V51606) and the USA (ASTM D6751). However, despite the studies focusing on the functional potential of *S. tonkinensis* seeds, it has not been given enough attention to the knowledge of the comprehensive biochemical composition.

Plant seed, as an embryonic plant formed on the mother tree, is very important for the reproduction of spermatophytes. Seed formation is determined by a series of metabolic processes of nutrient accumulation related to sugar, starch, lipid, amino acids, protein and other secondary metabolites with large species-specific difference [8,9,10]. There are many important metabolic pathways involved in seed development, e.g., amino acids metabolism, starch and sucrose metabolism, FA and flavonoids biosynthesis [11,12]. Understanding the active pathways involved in *S. tonkinensis* seed development requires approaches of metabolomics.

Metabolomics, as an alternative to genomics, transcriptomics and proteomics, plays an important role in studying the relationship between genotypes and phenotypes through related analyses of metabolites and pathway enrichment during plant development [13]. Metabolite fingerprinting can be conducted with gas chromatography-mass spectrometry (GC-MS) and liquid chromatography-mass spectrometry (LC-MS), the former approach is widely used for analyzing volatile organics and the latter for larger, more polar molecules [14,15]. Therefore, active metabolites and related pathways have been studied to assess metabolism levels in developing seeds, such as *Thlaspi arvense* L. and *Dipteryx alata* Vog. [16,17], which can partially provide the basis for studying in *S. tonkinensis*.

In this work, we analyzed samples from four stages during *S. tonkinensis* kernel development to evaluate metabolite fingerprinting and pathway enrichment by both GC-MS and LC-MS. The methodology used allowed us to understand the biochemical basis for developing *S. tonkinensis* kernels by (i) identifying metabolites related to main nutritional components biosynthesis, (ii) performing classification and change trends of identified metabolites, and (iii) confirming major metabolic pathways based on differential metabolites identification and pathway enrichment in developing kernels.

## 2. Results

### 2.1. Quality Control Result

**Figure 1:**
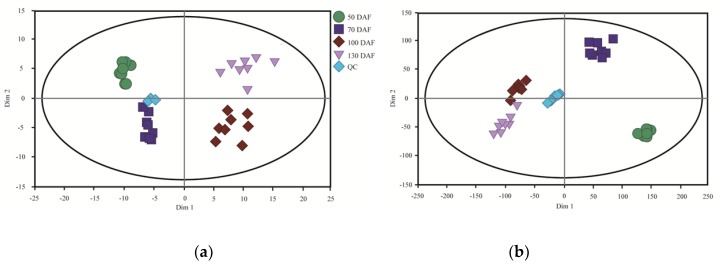
Quality control of samples from biological material. (**a**) Principal component analysis (PCA) of gas chromatography-mass spectrometry (GC-MS) analysis. (**b**) PCA of liquid chromatography-mass spectrometry (LC-MS) analysis.

The samples from 50, 70, 100 and 130 days after flowering (DAF) were analyzed through the GC-MS and LC-MS methods. Total ion currents were obtained after metabolomics analyses. These mass spectrograms were uniform at chromatographic peak, abundant in number and good in shape, without undesirable phenomena such as over-saturation or tail dragging which indicated good methods and the high quality of samples. The obtained metabolomics data of the GC-MS and LC-MS analyses adhered to a normal distribution, and a subsequent principal component analysis (PCA) showed high repeatability across biological replicates, as samples from four time points were distributed into four regions in a counter-clockwise sense except one from 100 DAF in LC-MS analysis (Figure 1). Hierarchical cluster analysis (HCA) showed similar result to the PCA analysis, with all of the eight biological replicates from each time point being clustered together (Figure S1).

### 2.2. Metabolite Identification

**Figure 2:**
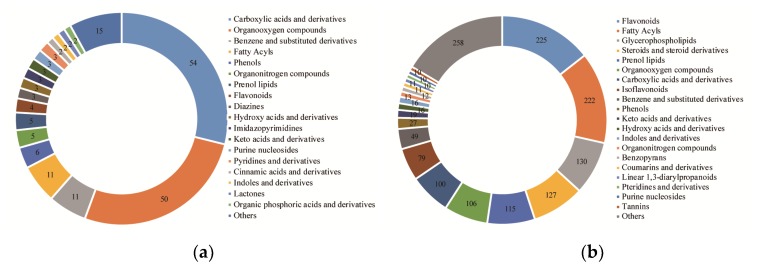
A classification of the metabolites during developing *S. tonkinensis* kernels. (**a**) The clustering results based on the metabolites of GC-MS analyses. (**b**) The clustering results based on the metabolites of LC-MS analyses.

**Figure 3:**
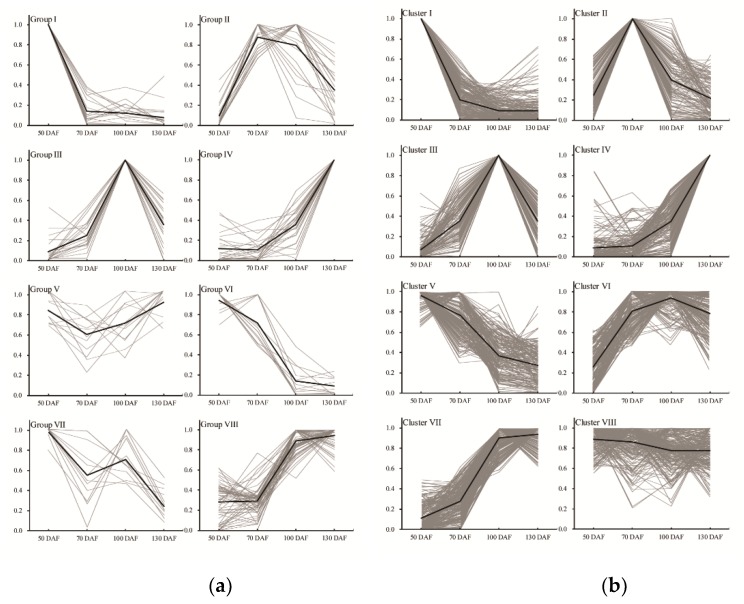
The *K*-means clustering of the metabolites during developing *S. tonkinensis* kernels. (**a**) The clustering results based on the metabolites of GC-MS analyses. (**b**) The clustering results based on the metabolites of LC-MS analyses.

A total of 187 metabolites were identified by GC-MS analysis in developing *S. tonkinensis* kernels (Table S1), which were grouped into 19 classes (Figure 2a). There were 54 metabolites (28.88%) classified into carboxylic acids and derivatives class, which mainly contained amino acids. The total amino acid concentration showed an up-down change trend peaking at 100 DAF. Alanine, glutamine, tryptophan, tyrosine and valine were the five most abundant amino acids across *S. tonkinensis* kernel development, representing between 74% and 95% of the total amino acids. Fifty metabolites (26.74%) were clustered into organooxygen compounds class with the major sub-class of the carbohydrates and carbohydrate conjugates, following the alcohols and polyols. Sucrose and glucose were the main soluble sugars, while inositol was the main sugar alcohol in developing *S. tonkinensis* kernels. Fatty acyls and benzene and substituted derivatives classes had 11 metabolites (5.88%), respectively. These 187 metabolites were clustered into eight groups based on their change patterns (Figure 3a). Groups I, II, III, and VI containing 19, 21, 20 and 25 metabolites, respectively, represented the metabolites with peak abundant levels at 50, 70, 100, and 130 DAF. Group V contained 13 metabolites with low abundant levels at 70 DAF. Groups VI and VII, containing 13 and 12 metabolites, respectively, represented metabolites with downward trends during all kernel development with a 10-day phase in the last stage or middle stage. Group VIII contained 64 metabolites, representing metabolites with lower abundant levels in the late stage (100 and 130 DAF) than in the early stage (50 and 70 DAF).

A total of 1556 metabolites were identified by LC-MS analysis in developing *S. tonkinensis* kernels (Table S2), which were grouped into 21 classes (Figure 2b). The most abundant class was the flavonoids class (225 metabolites, 14.37%), followed by the fatty acyls (222 metabolites, 14.18%), glycerophospholipids (130 metabolites, 8.30%), steroids and steroid derivatives (127 metabolites, 8.11%) and prenol lipids (115 metabolites, 7.34%). The metabolites in flavonoids class were mainly formed as flavans (FV, e.g., glicoisoflavanone), flavonoid glycosides (FG, e.g., quercetin 3, 7, 4’-*O*-triglucoside) and *o*-methylated flavonoids (OMF, e.g., 3, 4’, 5-trihydroxy-3’, 7-dimethoxyflavanone), which peaked at 50, 70 and 100 DAF, respectively. Among them, FG, composed of most kinds of metabolites, accounted for the major proportion but with a downward trend (dropping from 84.08% to 43.67%) during *S. tonkinensis* kernel development. Besides, OMF showed an upward trend from 9.95% to 30.20%, while FV increased from 2.40% to 23.70%. For fatty acyls class, there were four major free FAs, i.e., palmitic acid (C16:0), stearic acid (C18:0), oleic acid (18:1) and linoleic acid (C18:2). The FAs of C16:0 and C18:0 showed downward trends, while C18:1 and C18:2 had upward trends during *S. tonkinensis* kernel development. There were nine sub-classes in the glycerophospholipids class, i.e., cardiolipin (CL), cyclic lysophosphatidic acid (CPA), lysophospholipid (LyP), phosphatidic acid (PA), phosphatidylcholine (PC), phosphatidylethanolamine (PE), phosphatidylglycerol (PG), phosphatidylinositol (PI) and phosphatidylserine (PS). Among them, LyP accounted for the largest proportion with a down-up change trend, following PI with an up-down trend. The change in steroids and steroid derivatives class showed a downward trend overall during *S. tonkinensis* kernel development, with a major sub-class of steroidal glycosides. The content of prenol lipid remained stable with three major sub-classes of diterpenoids, monoterpenoids, and terpene glycosides. According to their change trends, these 1556 metabolites were classified into eight groups (Figure 3b). Clusters I, II, III, and IV contained 241, 147, 124 and 166 metabolites, respectively, representing the metabolites with peaking abundances at 50, 70, 100, and 130 DAF. Cluster V, containing 188 metabolites, represented metabolites that showed similar change trends to metabolites in group VI, having continuous downward trends throughout kernel development. Cluster VI contained 193 metabolites, representing metabolites with lower levels at the initial stage. Cluster VII, containing 238 metabolites, represented metabolites with high expression levels in the late stage (100 and 130 DAF). Cluster VIII, containing 269 metabolites, represented metabolites having somewhat lower expression levels in the late stage (100 and 130 DAF) than in the early stage (50 and 7 DAF).

For the classification and clustering of different metabolites (Tables S1 and S2), carboxylic acids and derivatives were identified by both LC-MS (100) and GC-MS (54), as they had similar change trends with high levels in late stages within group VIII and cluster VII. Organooxygen compounds were identified by both LC-MS (106) and GC-MS (50), and their abundance showed high levels in the early stage (group I and cluster I) or late stage (group VIII and cluster VII). Benzene and substituted derivatives were mainly identified through GC-MS (11), showing high levels in later stages (100 and 130 DAF) within group VIII. Flavonoids were mostly identified through LC-MS, and their abundance showed many obvious change trends within clusters I (45), IV (41), VI (33) and VII (37). Fatty acyls were also mainly identified by LC-MS, and had high levels in early stages within clusters I (32), II (26), III (27), V (30), and VIII (46). And, glycerophospholipids (130) and steroids and steroid derivatives (127) were mainly identified by LC-MS.

### 2.3. Differential Metabolites Analysis and Pathway Enrichment

**Figure 4:**
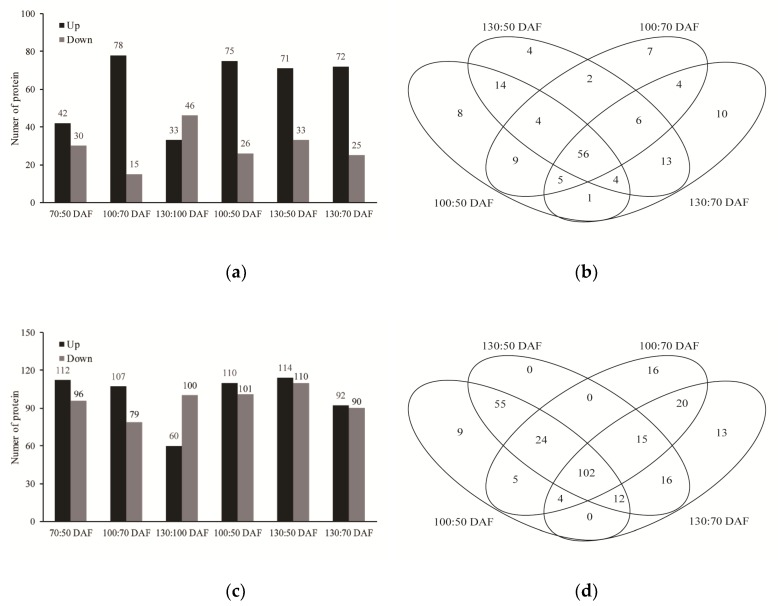
Statistical analysis of differential metabolites (DMs) during developing *S. tonkinensis* kernels. (**a**) The number of up-regulated and down-regulated DMs in the GC-MS analysis. (**b**) The distribution of DEPs at different time points in the GC-MS analysis. (**c**) The number of up-regulated and down-regulated DMs in the LC-MS analysis. (**d**) The distribution of DEPs at different time points in the LC-MS analysis.

Differential metabolites (DMs) between different time points were selected according to a VIP > 1 and a *p*-value < 0.05. Relative to each precious sampling point in GC-MS, a total of 72, 93, and 78 DMs were identified at 70, 100 and 130 DAF, respectively (Figure 4a). Relative to 50 DAF, a total of 101 and 103 DMs were identified at 100 and 130 DAF, respectively. There were 99 DMs identified between 70 and 130 DAF. For Venn diagrams of GC-MS analysis (Figure 4b), there were 56 DMs identified between the early stage (50 and 70 DAF) and late stage (100 and 130 DAF), and most of the DMs were upregulated. As same with the stander of GC-MS, DMs in LC-MS were identified to investigate changes in metabolism profile. Relative to each precious sampling point, a total of 208, 186 and 160 DMs were identified at 70, 100, and 130 DAF, respectively (Figure 4c). Relative to 50 DAF, a total of 211 and 224 DMs were identified at 100 and 130 DAF, respectively, while there were 182 DMs identified between 70 and 130 DAF. For Venn diagrams of LC-MS analysis (Figure 4d), the DMs were mainly concentrated (102 of the 358 DMs) between the early stage (50 and 70 DAF) and late stage (100 and 130 DAF), and the number of upregulated and downregulated was little different.

Based on the DMs identified during *S. tonkinensis* kernel development, Kyoto Encyclopedia of Genes and Genomes (KEGG) pathway enrichment was analyzed (Table S3). Alanine, aspartate and glutamate metabolism (ath00250), arginine and proline metabolism (ath00330), and glutathione metabolism (ath00480) were enriched between all sampling points of the DMs of both GC-MS and LC-MS analyses. Butanoate metabolism (ath00650), glycerolipid metabolism (ath00561), nitrogen metabolism (ath00910), the TCA cycle (ath00020), the pentose phosphate pathway (PPP) (ath00030), and pyrimidine metabolism (ath00240) were mainly enriched between all sampling points by DMs of GC-MS analysis. Biosynthesis of unsaturated fatty acids (ath01040), carbon fixation in photosynthetic organisms (ath00710), diterpenoid biosynthesis (ath00904), fatty acid biosynthesis (ath00061), flavonoid biosynthesis (ath00941), riboflavin metabolism (ath00740), and starch and sucrose metabolism (ath00500) were mainly enriched between the early stages (50 and 70 DAF) and late stages (100 and 130 DAF) of the LC-MS analysis.

## 3. Discussion

Metabonomic analysis is a direct, objective and accurate method to describe biological processes during plant development [18]. However, it needs effective quality control with complex multi-component samples to avoid the significant problems posed by unknown components [19]. In our study, we measured the change trends of metabolites in developing *S. tonkinensis* kernels through two metabolomics approaches, i.e., LC-MS and GC-MS analyses. The PCA and HCA showed high repeatability across biological replicates [20], with replicates from every time point being clustered together. In the PCA analysis, dim 1 may have been related to the time of kernel development from 50 DAF to 130 DAF, while dim 2 may have been related to the metabolic activity, with the beginning of nutrition accumulation around 70 DAF during *S. tonkinensis* kernel development [21].

During seed development, nutrients are transported into seeds and stored as nutritional components, e.g., amino acids, soluble protein, lipid, soluble sugar and starch [10]. Amino acids are important and active in developing kernels and are easily identified by GC-MS analysis [22]. The total amino acid content analyzed in GC-MS analysis showed an up-down trend with peaking at 100 DAF. A similar trend was also found in LS-MS analysis, which was consistent with the rapid increase in the total soluble protein content during kernel development [3]. Among the identified FAs, there were two major unsaturated FAs, i.e., C18:1 and C18:2, which showed continuously increasing trends and high proportions, with similar results in developing *Arachis hypogaea* L. kernels [23]. A high degree of unsaturated FAs reflects good fuel characteristics for bioresources [24], including density (ρ), kinematic viscosity (η), cetane number (CN), iodine value (IV), and cold filter plugging point (CFPP), which were analyzed in developing *S. tonkinensis* kernels by Wu et al. [6]. LyP, as an intermediate of triacylglycerol (TAG) biosynthesis [25], was the main form of metabolites in glycerophospholipids class (about 50%) with two major contents of lyP(18:1(9Z)) and lyP(18:2(9Z,12Z)). The main intracellular sugars and amino acids were found to be sucrose, glucose, tyrosine and glutamine, which were respectively common sources of carbon and nitrogen for developing plant embryos [17]. Furthermore, some phosphorylated metabolites (as key intermediates of glycolysis) of the PPP and TCA cycle (which is related to carbon metabolism) were identified, e.g., fructose-6-phosphate, fructose-1,6-bisphosphate, trehalose-6-phosphate and UDP-glucose [26], which showed high abundance levels at 70 and 100 DAF with rapid increases in lipid and starch biosynthesis during *S. tonkinensis* kernel development [21].

As bark produces the abundant benzoin used in incense, perfumes and medicines [27], *S. tonkinensis* has high economic and medicinal value [28,29] with abundant secondary metabolites. Flavonoids, as important plant secondary metabolites, are present in plant fruits, leaves and roots, such as *Polygonum minus* Huds. and *Mauritia flexuosa* L. [29,30]; and they can be found in many natural products beneficial to human health, such as teas and wines [31]. However, there are no studies that have focused on the secondary metabolites in *S. tonkinensis* kernels. In this study, we found 225 kinds of flavonoids through LC-MS in developing *S. tonkinensis* kernels. Among the flavonoids class, three main sub-classes, i.e., FG, FV and OMF accounted for up to 97.57% of flavonoids at 130 DAF, which could improve biological activities and pharmacological properties [32,33]. Otherwise, we found some metabolites classified into steroids and steroids derivatives, but the content decreased continuously to only 1/20 that of flavonoids. The identification of a high abundance of secondary metabolites in developing *S. tonkinensis* kernels may confirm that the species seeds have high medicinal value as its bark, like benzoin resin [1,34,35].

Metabolomics is an effective tool to assess the metabolites’ state during plant development [36]. Pathway analysis based on metabolites can provide insight into the complex biological processes of plant biomass biosynthesis, e.g., amino acids, organic acids, soluble sugar and protein [37]. In our study, the pathway enrichment mainly focused on three groups, such as pathways related to amino acids metabolism, carbon flow from sucrose to lipid and secondary metabolites biosynthesis, which are indications of protein, oil, and flavonoid accumulation in developing kernels, respectively [38,39]. Pathways related to amino acid metabolism were complex and involved in the biosynthesis of various kinds of amino acids, which play important roles in cellular processes [40]. Pathways related to carbon flow from sucrose to lipid, including glycolysis, the TCA cycle, the PPP, and FA biosynthesis, affect lipid accumulation in oilseeds [26]. The active pathway enrichment involved in amino acids and carbon flow was consistent with that protein (20%) and lipid (60%) are main nutrients in *S. tonkinensis* kernels, with perfect biodiesel properties for oil-seed resource [21]. Lipid content has a 40-day decrease during *S. tonkinensis* kernel development, which was considered as the result of carbon competition between oil and starch by Zhang et al. [3], but there is no effective evidence. Moreover, there are some gaps in the term of the total dry matter that needs to be studied, e.g., flavonoids and prenol lipid. The presence of various kinds of flavonoids and active pathway enrichment indicated that secondary metabolites account for a certain proportion in developing *S. tonkinensis* kernels and then may lead to a decrease in carbon flux to lipid around 100 DAF.

We analyzed the comprehensive biochemical composition by sampling at 50, 70, 100, and 130 DAF. The size and structure of *S. tonkinensis* kernel began to develop at 50 DAF, and lipid began to appear. Most nutrients in the kernels began to accumulate rapidly at 70 DAF with high enzyme activity, e.g., acetyl-CoA carboxylase, phosphoglucose isomerase, and the pyruvate dehydrogenase complex [3,21]. There was a continuously decreasing trend in lipid content between 80 and 120 DAF, which may have been related to the biosynthesis of flavonoids analyzed in the present study rather than the carbon competition between oil and starch. The seeds matures at 130 DAF, which is close to the best harvest time to get *S. tonkinensis* seed oil of better quantity and quality for biodiesel. Overall, the time points selected in this study represented four important turning points of kernel development, nutrients accumulation, flavonoids accumulation and kernel harvest.

## 4. Materials and Methods

### 4.1. Plant Materials

Forty *S. tonkinensis* plants were used in this study, which were planted in the Styracaceae Germplasm Repository located in the Luhe District of Nanjing, China (32°32′ N, 118°50′ E). Trees flowered in late May and were tagged for sampling later. According to our previously published data [3,21] on the morphological and physiological changes of developing *S. tonkinensis* kernels, we selected samples from four time points in order to explore changes in the metabolome, i.e., 50 DAF (the previous stage before seed matter rapid increase), 70 DAF (during the aforementioned steep rise in nutrient content), 100 DAF (the stage with decreasing oil content and increasing starch content) and 130 DAF (the final maturation stage). Fresh fruits were randomly collected and immediately transferred to the laboratory on dry ice with eight biological replicates at each time point. The pericarp and seed coat were removed, and the kernels were immediately frozen in liquid nitrogen and stored at −80 °C for metabolomics analysis.

### 4.2. GC-MS Analysis

#### 4.2.1. GC-MS Sample Preparation

1. 60 mg samples were transferred into a 1.5 mL Eppendorf tube with two small steel balls.
2. 40 μL boc-2-chloro-L-phenylalanine in 0.3 mg/mL methanol solution as internal standard and 360 μL pre-cooling methanol were added respectively.
3. The samples were placed at −80 °C for 2 min and then ground at 60 Hz for 2 min, before ultrasonic extraction at room temperature for 30 min.
4. 200 μL chloroform and 400 μL water were added.
5. The samples were extracted at room temperature for 30 min and then centrifuged at 12,000 rpm at 4 °C for 10 min.
6. 500 μL supernatant was transferred into GC vials.
7. The samples were dried with a freeze concentration centrifugal dryer, and then 80 μL methoxamine-hydrochloride in pyridine (15 mg/mL) was added (with vortex oscillation for 2 min).This was placed at 37 °C for 90 min for an oximation reaction.
8. 80 μL bis (trimethylsilyl) trifluoroacetamide (BSTFA) containing 1% trimethylchlorosilane (TMCS) and 20 μL n-hexane were added, and vortex oscillation for 2 min followed.
9. The samples were placed at 70 °C for 60 min and then at room temperature for 30 min for GM-LC analysis.
10. Quality control samples (QCs) were prepared by mixing all samples, which were analyzed using the same method as with the analytic samples. The QCs were injected at regular intervals to assess the repeatability of the whole analytical process.

#### 4.2.2. GC Conditions and MS Method

The GC-MS analysis was performed on an Agilent 7890A/5975C GC-MS system (Agilent, Santa Clara, CA, USA) equipped with two split/splitless inlets and a multi-purpose sampler using a HP-5MS column (30 m × 0.25 mm × 0.25 μm; Agilent J&W Scientific, Folsom, CA, USA). Refer to previous studies [17,41] for the appropriate modifications during the separation process.

The injection volume of the sample was 1 μL and the injector temperature was 260 °C. The carrier gas was helium at a flow of 1 mL/min. The GC temperature was initially 60 °C for 2 min, and then it gradually increased at 8 °C/min to 310 °C, which was held for 6 min.

An electro ionization system was used with 70-eV of ionization energy. The mass spectra of eluted compounds were recorded over an *m/z* range of 50–600.

#### 4.2.3. GC-MS Data Collection

The GC-MS raw data were analyzed by Chroma TOF v4.34 software (LECO, Stockport, UK). After removing the internal standard peak and any known false positive peaks, e.g., including noise, column loss and derivative peaks, the GC-MS data were obtained with three-dimensional datasets including sample information, metabolism names, and peak intensities.

### 4.3. LC-MS Analysis

#### 4.3.1. LC-MS Sample Preparation

1. 80 mg samples were transferred into a 1.5 mL Eppendorf tube with two small steel balls.
2. 20 μL boc-2-chloro-L-phenylalanine in methanol and water (1/1, *v/v*) as internal standard and 1 mL pre-cooling methanol solution (7/1, *v/v*) were added respectively.
3. The samples were placed at −80 °C for 2 min and then ground at 60 Hz for 2 min, before ultrasonic extraction at room temperature for 30 min.
4. The samples were placed at 4 °C for 20 min and then centrifuged at 14,000 rpm at 4 °C for 10 min.
5. 500 μL supernatants were filtered through 0.22 μm microfilters and then transferred into LC vials.
6. QCs were prepared by mixing all of the all samples in the same way as with the GC-MS analysis.

#### 4.3.2. LC Conditions and MS Method

The LC-MS analysis was performed on an Ultimate 3000-Velos Pro system (Dionex, Sunnyvale, CA, USA) equipped with a binary solvent delivery manager and a multi-purpose sampler, coupled with a Q exactive orbitrap mass spectrometer (Thermo Fisher Scientific, Waltham, MA, USA).Refer to previous studies [42] for the appropriate modifications during the separation process.

The LC separation was performed using a BEH C18 column (100 nm × 2.1 mm I.D., 1.7 μm; Waters, Milford, CT, USA) at a flow rate of 0.4 mL/min and a column temperature of 45 °C.

During the LC process, mobile phase A was water with 0.1% formic acid (*v/v*) and mobile phase B was acetonitrile containing 0.1% formic acid (*v/v*).

The separation was performed using the following gradient: going from 5% to 25% B in 1.5 min, from 25% to 100% in 8.5 min, at 100% for 3 min, then declining from 100% to 5% in 0.5 min and at 5% for 1 min.

The MS data were collected using an LTQ Orbitrap Mass Spectrometer (Thermo Fisher Scientific, Waltham, MA, USA) equipped with an electrospray ionization (ESI) source operated in either positive or negative ion mode. The capillary and source temperature were set at 350 °C, with a desolvation gas flow of 45 L/h. Centroid data were collected from 50 *m/z* to 1000 *m/z* with a 30,000 resolution.

#### 4.3.3. LC-MS Data Collection

The LC-MS raw data were analyzed by Progenesis QI v2.0 software (Waters Corporation, Milford, CT, USA) with a retention time (RT) range of 0.5–14 min, a mass range of 50–1000 Da, and a mass tolerance of 0.01 Da. Isotopic peaks were excluded from the analysis, the noise elimination level was set at 10, the minimum intensity was set to 15% of base peak intensity, and finally, the RT tolerance was set at 0.01 min. The LC-MS data were obtained with three-dimensional datasets included *m/z*, peak RT, and peak intensities, and RT-*m/z* pairs were used as the identifier for each ion.

### 4.4. Data Analysis and Bioinformatics Analysis

The GC-MS and LC-MS data were imported into the SIMCA-P + 14.0 software package (Umetrics, Umeå, Sweden) for statistical analysis [37]. A PCA, HCA and (orthogonal) partial least-squares-discriminant analysis ((O)PLS-DA) were carried out to visualize the metabolic alterations among experimental groups, after mean centering and unit variance scaling. Variable importance in the projection (VIP) ranks the overall contribution of each variable to the (O)PLS-DA model, and those variables with VIP > 1.0 were considered relevant for group discrimination. In this study, a default seven-round cross-validation was applied, with one-seventh of the samples being excluded from the mathematical model in each round in order to guard against overfitting. The identified metabolites were grouped by their biochemical properties and change patterns (www.omicsolution.org). The differential metabolisms were identified by (O)PLS-DA if their VIP was over 1 with a *p*-value < 0.05. And then the pathway enrichment according to the identified differential metabolisms was analyzed using KEGG databases [43].

## 5. Conclusions

In our study, we collected samples from 50, 70, 100, and 130 DAF to explore spatiotemporal comprehensive biochemical composition. It’s the first time to identify flavonoids class in *S. tonkinensis* kernels, which were mainly formed as flavans, flavonoid glycosides and *o*-methylated flavonoids. All of the metabolites were grouped based on their biochemistry properties and change trends. The major metabolite classes were focused on carboxylic acids and derivatives, flavonoids, fatty acyls, glycerophospholipids, organooxygen compounds, prenol lipids, and steroids and steroid derivatives. Alanine, glutamine, tryptophan, tyrosine, and valine were the top five most abundant amino acids; and palmitic acid, stearic acid, oleic acid, and linoleic acid were four major free FAs. Pathway enrichment was mainly focused on three groups, amino acids metabolism, carbon flow from sucrose to lipid and secondary metabolites biosynthesis. *S. tonkinensis* seeds have application value in biofuels and medicines with high contents lipid and flavonoids.

## Supplementary Materials

The following are available online at https://www.mdpi.com/2218-1989/10/1/21/s1, Figure S1: The results of the hierarchical cluster analysis (HCA) in the GC-MS and LC-MS analyses. (**a**) HCA analysis in GC-MS. (**b**) HCA analysis in LC-MS, Table S1: The list of metabolites identified by GC-MS analysis, Table S2: The list of metabolites identified by LC-MS analysis, Table S3: The pathway enrichment analysis based on differential metabolites.
