# Supplementary material for: Metabolite Profiling and Classification of Developing Styrax tonkinensis Kernels
Source: Metabolites. 2020 Jan 1;10(1):21. doi: 10.3390/metabo10010021 (PMC7022597; doi:10.3390/metabo10010021)
Supplement: Supplementary file 1 [file metabolites-10-00021-s001.zip › Figure S1.pdf]

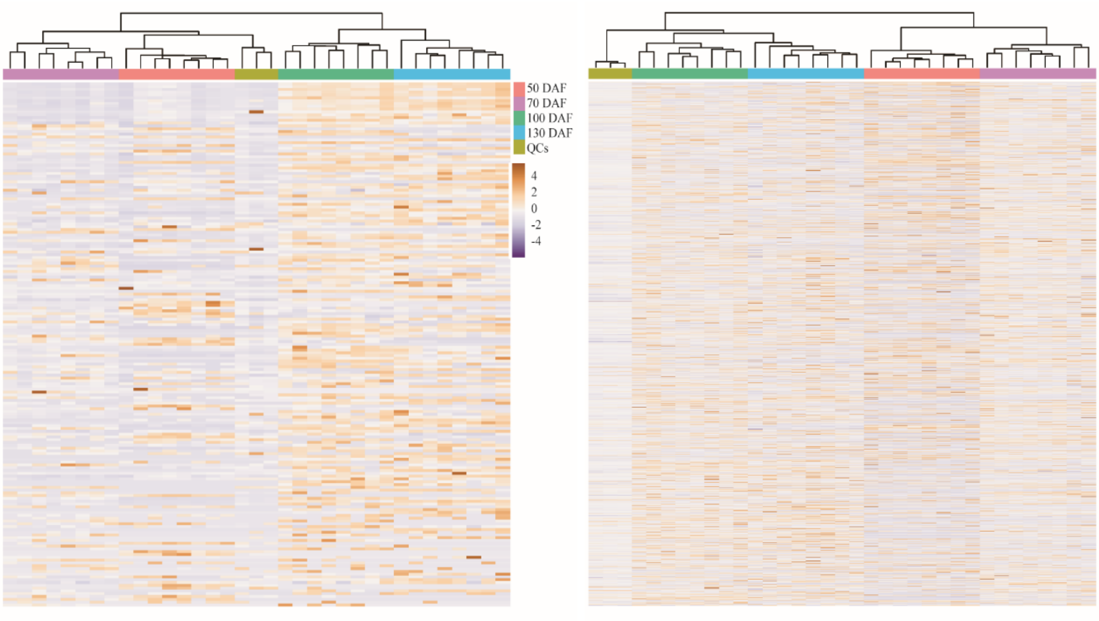

(**a**) (**b**)

Figure S1. The result of hierarchical cluster analysis (HCA) in GC-MS and LC-MS analyses. (A) HCA analysis in GC-MS. (B) HCA analysis in LC-MS
